# FXR activation reduces the formation of macrophage foam cells and atherosclerotic plaque, possibly by down regulating hepatic lipase in macrophages

**DOI:** 10.1002/2211-5463.13925

**Published:** 2024-11-27

**Authors:** Qiang Gu, Jia Liu, Li Li Shen

**Affiliations:** ^1^ Institute of Cardiovascular Surgery, Xinqiao Hospital Second Affiliated Hospital of the Army Military Medical University Chongqing China; ^2^ Department of Pathology Chongqing University Cancer Hospital China; ^3^ Chongqing Key Laboratory for Intelligent Oncology in Breast Cancer (iCQBC) Chongqing University Cancer Hospital China

**Keywords:** atherosclerotic plaque, Farnesoid X receptor, hepatic lipase, macrophages

## Abstract

Macrophages are the most important immune cells affecting the formation of atherosclerotic plaque. Nevertheless, the mechanisms that promote formation of foamy macrophages during atherogenesis remain poorly understood. This study explored the effects of Farnesoid X receptor (FXR) and hepatic lipase (HL, encoded by *LIPC*) on atherogenesis, particularly in foamy macrophage formation. A luciferase reporter assay indicated that FXR could bind to the *LIPC* promoter and inhibit *LIPC* transcription. FXR agonist GW4064 decreased HL expression, foam cell formation, and increased the expression of FXR downstream genes and polarization to M2 in ox‐LDL‐induced THP‐1 and U937 foam cells. In addition, GW4064 exerted anti‐atherosclerotic effects in ApoE^−/−^ mice, manifested as decreased serum cholesterol and triglyceride levels, and alleviated atherosclerotic plaque formation. Collectively, FXR exerted anti‐atherosclerotic effects, possibly by negatively regulating HL expression in macrophages.

## Graphical abstract

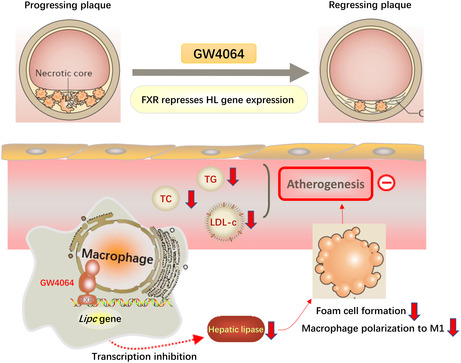

**Abbreviations**
FXR: Farnesoid X receptor
HDL: high‐density lipoprotein
HL: hepatic lipase
LDL: low‐density lipoprotein
LDL‐C: low‐density lipoprotein‐cholesterol
NR: nuclear receptor
ORO: Oil Red O
SMPD3: sphingomyelin phosphodiesterase 3
TC: total cholesterol
TG: triglyceride

Atherosclerosis, a chronic inflammatory disease of the arterial wall, represents a major global health concern due to its association with adverse cardiovascular events, such as heart attack and stroke. The disease process involves the accumulation of lipids, inflammatory cells, and fibrous elements within the artery wall, resulting in the formation of atherosclerotic plaque [1]. These plaques can cause narrowing of the blood vessel lumen, which limits blood flow. In extreme cases, the plaques can rupture, leading to vessel occlusion and tissue ischemia. Reports suggest that macrophages are the most important immune cells affecting the formation of atherosclerotic plaque [2, 3]. However, the mechanisms that promote formation of foamy macrophages during atherogenesis are poorly understood.

Among the key cellular players in atherogenesis, foamy macrophages have emerged as crucial mediators. Abnormal levels of serum low‐density lipoprotein (LDL) and cholesterol often affect macrophage polarization and activation through lipoproteins [4]. In addition, numerous genes in macrophage cells become abnormally expressed due to a remission of hyperlipidemia and thus promote macrophage foam formation [5, 6, 7]. Understanding the molecular mechanisms underlying the formation of foamy macrophages is essential for developing effective therapeutic strategies against this debilitating disease.

Farnesoid X receptor (FXR) is a member of the nuclear receptor (NR) superfamily that is mainly expressed in the liver and small intestine. FXR plays an important role in bile acid, lipid, and glucose metabolism [8]. As a ligand‐activated transcription factor, FXR regulates the expression of various genes by binding to DNA in the form of monomers or heterodimers. A recent study reported that FXR could be activated by bile acids and significantly reduce lipid peroxidation by upregulating the expression of ferroptosis‐related genes [9]. Wu *et al*. [10] found that the intestinal expression level of FXR was involved in the development of atherosclerosis, and suppression of FXR could decrease atherosclerosis by regulating sphingomyelin phosphodiesterase 3 (SMPD3). In addition, FXR was found to promote the transfer of phospholipids between lipoproteins and regulate plasma high‐density lipoprotein (HDL) metabolism by modulating the transcription of phospholipid transfer protein genes [11]. However, whether FXR is abnormally expressed and what role it plays in the process of macrophage foam cell formation has not been reported.

Our preliminary studies found that an FXR agonist could inhibit the transcription of hepatic lipase (HL, encoded by *LIPC*) in a dose‐ and time‐dependent manner in human hepatoma HepG2 cells and inhibit HL activity [12]. HL plays a crucial role in lipid metabolism, and its activity is closely related to plasma lipoprotein levels [13, 14]. In recent years, studies have shown that abnormal hepatic lipase activity is closely related to the occurrence and development of atherosclerosis [15, 16, 17]. Hence, we attempted to explore whether FXR could regulate HL expression in macrophages.

This study explored the effects of FXR and HL on atherogenesis. Herein, we hypothesized that an FXR agonist might exert an anti‐atherosclerotic effect by downregulating hepatic lipase expression in macrophages, which could reduce the production of atherosclerotic plaque. We tested this hypothesis by conducting a series of *in vitro* experiments with U937 and THP‐1 cells, as well as by conducting *in vivo* experiments with ApoE^−/−^ mice.

## Materials and methods

### Cell culture and treatments

U937 and THP‐1 cells were purchased from the American Type Culture Collection (Manassas, VA, USA). The cells were cultured in RPMI 1640 medium (Gibco, Grand Island, NY, USA) supplemented with 10% fetal bovine serum (Hyclone, Logan, UT, USA), and maintained at 37 °C in an incubator containing 5% CO_2_. U937 and THP‐1 cells were induced to form foamy macrophages and then treated with increasing concentrations of GW4064 (0, 0.02, 0.2, or 2 nmol·L^−1^, respectively) for 48 h.

### Luciferase report experiments

A luciferase reporter experiment was conducted to detect the binding sequence of FXR on the *LIPC* promoter. The human *LIPC* proximal promoter segment from −1900 to +100 bp in U937 cells was constructed using PCR. Next, the fragments were reorganized to form the pGL3‐Basic vector (5′KpnI and 3′ Hind III; Promega, Madison, WI, USA), and named as pGL3/HL/−1900 ~ +100 bp, to generate HL (−1900/+100 bp)‐luc. A series of nested deletions were generated using HL (−1900/+100 bp)‐luc as a template. The FXR‐binding sites on the human HL promoter are shown in Table S1. Next, the luciferase reporter and FXR overexpression plasmid were transfected into U937 cells and luciferase activity was detected. Briefly, cells grown in 96‐well plates were transiently transfected with the indicated constructs (0.2 mg per well) and pGL3/HL/ER10‐Renilla (0.01 mg per well) for 24 h by using Jet PEI transfection reagent according to the manufacturer's instructions. The viability of cells was > 90%, as determined under a high magnification microscope. After 24 h, the cells were harvested for the luciferase activity assay, which was performed using a Dual‐Luciferase Reporter Assay System (Promega). Next, a second luciferase reporter experiment was performed to confirm the binding sequence. Point mutations were introduced into the binding sequence by using a QuikChange Site‐Directed Mutagenesis Kit (Stratagene, San Diego, CA, USA) according to manufacturer's instructions (boldface letters indicate transcription factor binding regions, and lowercase letters show mutated bases), and the sequence was reorganized to create the pGL3‐Basic luciferase reporter. Next, the wild‐type reporter and mutant reporter were transfected into U937 cells, respectively, along with the FXR overexpression plasmid or NC vector. Luciferase activity was measured using identical methods.

### Foam cell formation

THP‐1 and U937 cells were incubated with 100 ng·mL^−1^ of PMA (MedChemExpress, Monmouth Junction, NJ, USA) for 48 h to induce macrophage differentiation. To induce foam cell formation, THP‐1 and U937 macrophages were incubated with 80 μg·mL^−1^ ox‐LDL (Yiyuanbiotech, Shanghai, China) for 48 h, with or without GW4064. Foam cell formation was evaluated by Oil Red O (ORO) staining.

### Flow cytometry

To confirm the effect of GW4064 on macrophage polarization, flow cytometry was used to detect the M1 biomarker CD68 and the M2 biomarkers CD206. Briefly, cells were washed and separately stained with anti‐CD86 (GTX02615; GeneTex, Irvine, CA, USA) or anti‐CD206 (GTX03378; GeneTex) for 10 min; after which, they were incubated with fluorescent antibodies for 30 min. Finally, the positive cells were analyzed by flow cytometry and the data were analyzed using flowjo software (Becton, Dickinson and Company, New Jersey, USA).

### ELISA

The levels of IL‐6 (an M1 marker) and IL‐10 (an M2 marker) in cell culture supernatants were detected using ELISA kits (PI330 and PI528; Beyotime, Shanghai, China). Briefly, the cells were centrifuged and the supernatants were used to detect the markers according to the manufacturer's instructions. The absorbance of each culture plate well at 450 nm was measured with a microplate reader and used to calculate the levels of cytokines.

### Chromatin immunoprecipitation assay (ChIP)

U037 cells were cultured with or without 2 nmol·L^−1^ GW4064 and analyzed using a ChIP Assay kit (Millipore, Billerica, MA, USA). 5 × 10^6^ macrophages were washed with PBS and then fixed in 1% formaldehyde for 10 min at room temperature. The fixed cells were harvested, lysed, and sonicated for 10 cycles of 10 s on/20 s off and 50% AMPL with a Sonics VCX 130 sonicator (Sonics & Materials Inc, Newtown, CT, USA). An antibody directed against FXR (sc‐25309; 5 mg per 1 mg total protein) and antibodies directed against IgG (sc‐66931; 10 mL per 1 mg total protein) were obtained from Santa Cruz Biotechnology (Dallas, TX, USA). The precipitated DNA was subjected to PCR amplification. The primer sequences used for amplification were as follows: forward, 5′‐GCCACGTGGAAGCCACCTA‐3′; reverse, 5′ CTTATCCTGACACATTTTGAG‐3′.

### Electrophoretic mobility shift assay (EMSA)

Nuclear extracts were prepared using NE‐PER nuclear extraction reagent (Pierce, Rockford, IL, USA). DNA probes containing the FXR site of the HL promoter and the corresponding primers are listed in Table S2. The DNA‐binding reaction was performed using a Light shift Chemiluminescent EMSA Kit (Pierce). A 50‐fold molar excess of non‐labeled oligonucleotide was simultaneously added as a competitor with the labeled probe. To identify DNA‐binding proteins, nuclear extracts were incubated with 3 mg of antibody directed against FXR (sc‐25309; Santa Cruz Biotechnology) or control IgG (sc‐66931; Santa Cruz Biotechnology) at room temperature for 20 min before the labeled probe was added.

### Animals and treatments

C57BL/6 mice (6‐week‐old males, *n* = 10) were used as wild‐type control animals. The mice were housed in a controlled environment and fed standard laboratory rodent chow (NFD, normal‐fat diet) throughout the study period. Male 6‐week‐old C57BL/6 mice (*n* = 10) and ApoE^−/−^ mice (*n* = 30) were purchased from Beijing HFK Bioscience Company (Beijing, China). To ensure development of high‐fat‐induced atherosclerosis, the ApoE^−/−^ mice were fed a HFD (high‐fat diet: standard laboratory rodent chow supplemented with 1% cholesterol, 10% fat, and 0.2% bile salt) for 7 weeks. The high‐fat diet food was obtained from the Army Medical University Laboratory Animal Center. After establishing the mouse model of atherosclerosis, the ApoE^−/−^ mice were randomly assigned to the following three groups (10 mice per group): (a) ApoE^−/−^ control group (without GW4064，*n* = 10); low‐dose GW4064 group (10 mg·kg^−1^, *n* = 10); high‐dose GW4064 group (20 mg·kg^−1^, *n* = 10). Normal male C57 BL/6 mice with the same genetic background and same age were used as control animals. Mice receiving the FXR agonist (GW4064) were gavaged twice a week for 8 weeks, and the body weight of each mouse was measured and recorded weekly beginning at 6 weeks of age. All mice were housed in a specific pathogen‐free facility, and no mouse was excluded from the final analysis. Finally, the mice were fasted for 12 h and then anesthetized with pentobarbital sodium. The levels of TC (total cholesterol), TG (triglyceride), and LDL‐C (low‐density lipoprotein‐cholesterol) were measured by enzymatic assays (Wako Chemicals, Osaka, Japan). Peritoneal‐derived macrophages were obtained from wild‐type and ApoE^−/−^ mice. The animal experimental procedures were reviewed and approved by the Institutional Animal Care and Use Committee of Army Medical University (AMUWEC20187017).

### RNA analysis

Quantitative reverse transcription PCR was performed as previously described [12, 18]. Real‐time qPCR for HL mRNA was performed using synthetic TAQMAN gene‐specific primers (Applied Biosystems, Waltham, MA, USA), and under the following conditions: denaturation, annealing, and extension at 94 °C, 55 °C, and 72 °C for 30 s, 30 s, and 1 min, respectively, for 40 cycles. The primer sequences used for RNA analysis are listed in Table S3. GAPDH served as an internal control. Finally, gene expression was quantitated using the $2^{-\Delta \Delta C_{t}}$ method.

### Immunohistochemistry (IHC)

Antigens were retrieved from tissue sections by incubating the sections overnight at 4 °C in citrate buffer while blocking endogenous peroxidase activity with 3% H_2_O_2_. Next, the sections were incubated with primary antibodies against HL (ab175105; Abcam, Cambridge, UK), followed by incubation with Goat Anti‐Rabbit IgG H&L (HRP) (ab7090; Abcam) at room temperature for 1 h. Finally, the sections were observed under a microscope and photographed.

### Oil Red O (ORO) staining

Lipid accumulation in THP‐1 macrophages treated with ox‐LDL was detected by ORO staining as previously described [19]. Briefly, cells were washed twice with PBS and fixed with 10% formalin in PBS for 1 h; after which, they were washed an additional three times with water and dried. Next, the cells were stained with ORO (six parts of saturated ORO dye in isopropanol and four parts of water) for 15 min. Excess stain was removed by washing with 70% ethanol, and the stained cells were then washed with water. Spectrophotometric quantitation of staining density was measured by imagej software (National Institutes of Health, Bethesda, USA).

### Tissue staining

Each mouse heart was perfused with ice‐cold phosphate‐buffered saline for 5 min, and the aortic sinus was cut into serial cryosections of 7‐μm thickness. Next, the heart and aorta were dissected, embedded in paraffin, and cut into 4 μm thick sections. *En face* lesions at the aortic arch were stained with Oil Red O (Sigma Aldrich, Shanghai, China), and cross‐sectional lesions in the aortic root were stained with H&E to evaluate the size of the atherosclerotic lesions. The areas of the cross‐sectional and *en face* lesions were outlined using image pro plus 6.0 software (Media Cybernetics, Silver Springs, MD, USA), and the positive areas were calculated. The atherosclerotic plaque at the aortic sinus was quantified by ORO staining of lipid deposits, as previously described [20]. The plaque area for each mouse was calculated as the mean of three sections. The *en face* lesions at the aortic arch were also observed by H&E staining. The sections were deparaffinized by immersion in xylene and then rehydrated in ethanol; after which, they were stained with hematoxylin for 5 min, with eosin for 3 min, dehydrated with ethanol and xylene, and finally observed under a light microscope.

### Western blot analysis

Equal amounts of protein (20 μg) were separated on 10% SDS‐gels and the protein bands were transferred onto PVDF membranes, which were subsequently blocked. The membranes were then incubated overnight with rabbit anti‐HL antibodies (DF6411; Affinity Biosciences, Cincinnati, OH, USA), anti‐Rarb antibodies (AF0249; Affinity Biosciences), anti‐Cyp26b1 antibodies (DF12194; Affinity Biosciences), anti‐iNOS (AF0199; Affinity Biosciences), anti‐Arg1 (DF6657; Affinity Biosciences), or anti‐GAPDH antibodies (AF7021; Affinity Biosciences) at 4 °C. Next, the membranes were incubated with HRP anti‐rabbit IgG for 2 h and then developed using electrochemiluminescence (ECL). The staining density of each protein band was quantified using imagej software and normalized to that of GAPDH.

### Statistical analysis

Each experiment was repeated at least three times, and the different cell preparations were analyzed in triplicate. Statistical results are shown as a mean value ± SD. The statistical significance of differences between groups was evaluated by using Student's *t*‐test or one‐way analysis of variance. A *P*‐value < 0.05 was considered to be statistically significant.

## Results

### FXR agonist‐mediated downregulation of human macrophage HL gene expression occurred at the transcriptional level

**Fig. 1:**
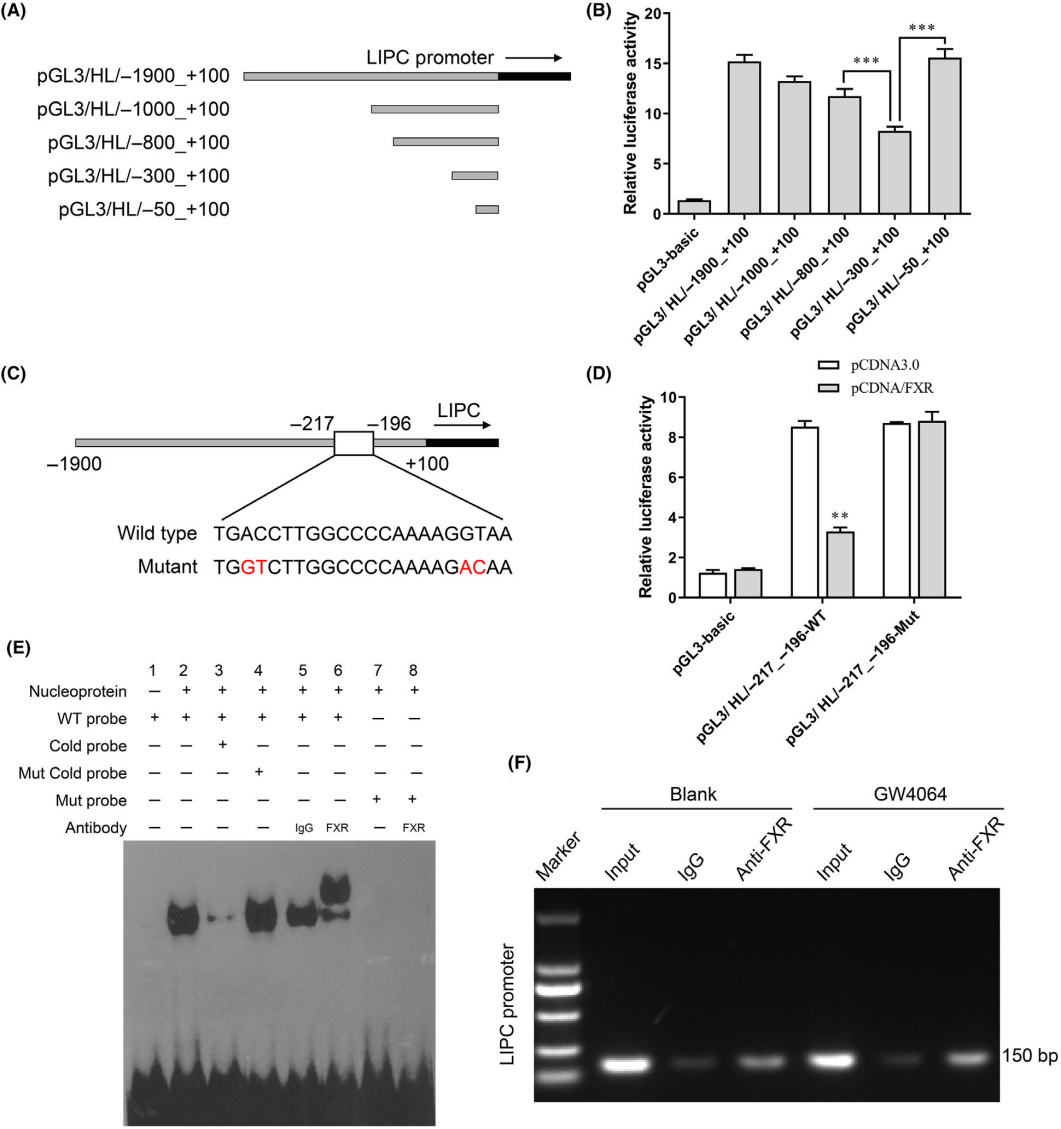
An FXR‐binding site located at −217 to −196 bp was conserved in a fragment of the human *HL* gene promoter. (A) U937 cells were transfected with full length or altered human HL promoter constructs. (B) Luciferase reporter assays were conducted using U937 cells transfected with the luciferase reporter pGL3 plasmid and containing various deleted fragments of the proximal human HL promoter. The cells were harvested for use in luciferase activity assays and the data were analyzed by one‐way analysis of variance. (C) A conserved FXR‐binding element in the human HL promoter (GenBank accession number NM_000236.2) was predicted by JASPAR. (D) The binding sequence was confirmed by a dual‐luciferase reporter gene assay (*t*‐test analysis). (E) The binding of FXR to the *LIPC* promoter was confirmed by an EMSA. (F) Lysates of human macrophage U937 cells with the indicated treatments were prepared for ChIP assays performed using anti‐FXR Ab or control IgG. ***P < 0.001; **0.001 < *P* < 0.01; value ± SD, Student's *t*‐test or one‐way analysis of variance; *n* = 3.

A sequence evaluation of the proximal *LIPC* gene promoter revealed the presence of FXR direct binding elements (Fig. 1A) located at −300 to +100 in the *LIPC* promoter (Fig. 1B). That analysis also revealed a FXR‐binding site conserved in humans located at −217 to 196 bp in a fragment of the human *LIPC* gene promoter (Fig. 1C), and a dual‐luciferase reporter assay further confirmed the binding sequence (Fig. 1D). Binding of FXR to the *LIPC* gene promoter elements was evaluated by an electrophoretic mobility shift assay (EMSA) that was conducted using nuclear extracts from human U937 cells (Fig. 1E). An EMESA using a 3′‐biotin‐labeled DNA probe targeted to the HL promoter revealed an apparent DNA‐protein complex after the nuclear extracts of U937 cells were added. Significant numbers of single DNA/protein complexes were detected with nuclear extracts from U937 cells when using the WT probe (lane 6) but not when using the Mut probe (lane 8). Binding between FXR protein and the HL promoter was also confirmed by CHIP experiments. As shown in Fig. 1F, the FXR antibody significantly enriched more LIPC promoter than IgG. Moreover, after treatment with GW4064, the LIPC promoter became further enriched by the FXR antibody. These findings suggested that FXR binding occurred in the region of the *LIPC* proximal promoter.

### Foam cell formation was induced by ox‐LDL

**Fig. 2:**
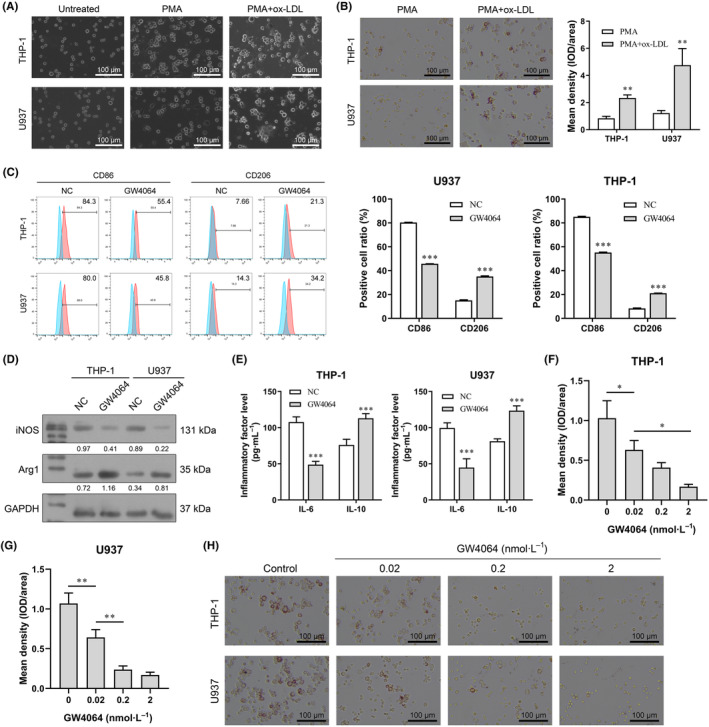
Effect of an FXR agonist on ox‐LDL‐induced macrophage‐derived foam cell formation. (A) Macrophages induced from THP‐1 and U937 cells by PMA were incubated with ox‐LDL for 48 h to induce foam cell formation. (B) Oil Red O staining was used to confirm the foam cell formation induced by ox‐LDL (*t*‐test analysis). (C) The effect of GW4064 on macrophage polarization was detected by flow cytometry (*t*‐test analysis). (D) The M1 macrophage marker iNOS and M2 macrophage marker Arg1 were detected by western blotting. (E) The M1 macrophage marker IL‐6 and M2 macrophage marker IL‐10 were detected by ELISA (*t*‐test analysis). (F–H) The concentration‐dependent effect of an FXR agonist (GW4064) on ox‐LDL‐induced foam cell formation was analyzed by detecting intracellular lipid accumulation by use of Oil Red O staining (one‐way analysis of variance). ***P < 0.001; **0.001 < *P* < 0.01; *P < 0.05; value ± SD, Student's *t*‐test or one‐way analysis of variance; *n* = 3.

**Fig. 3:**
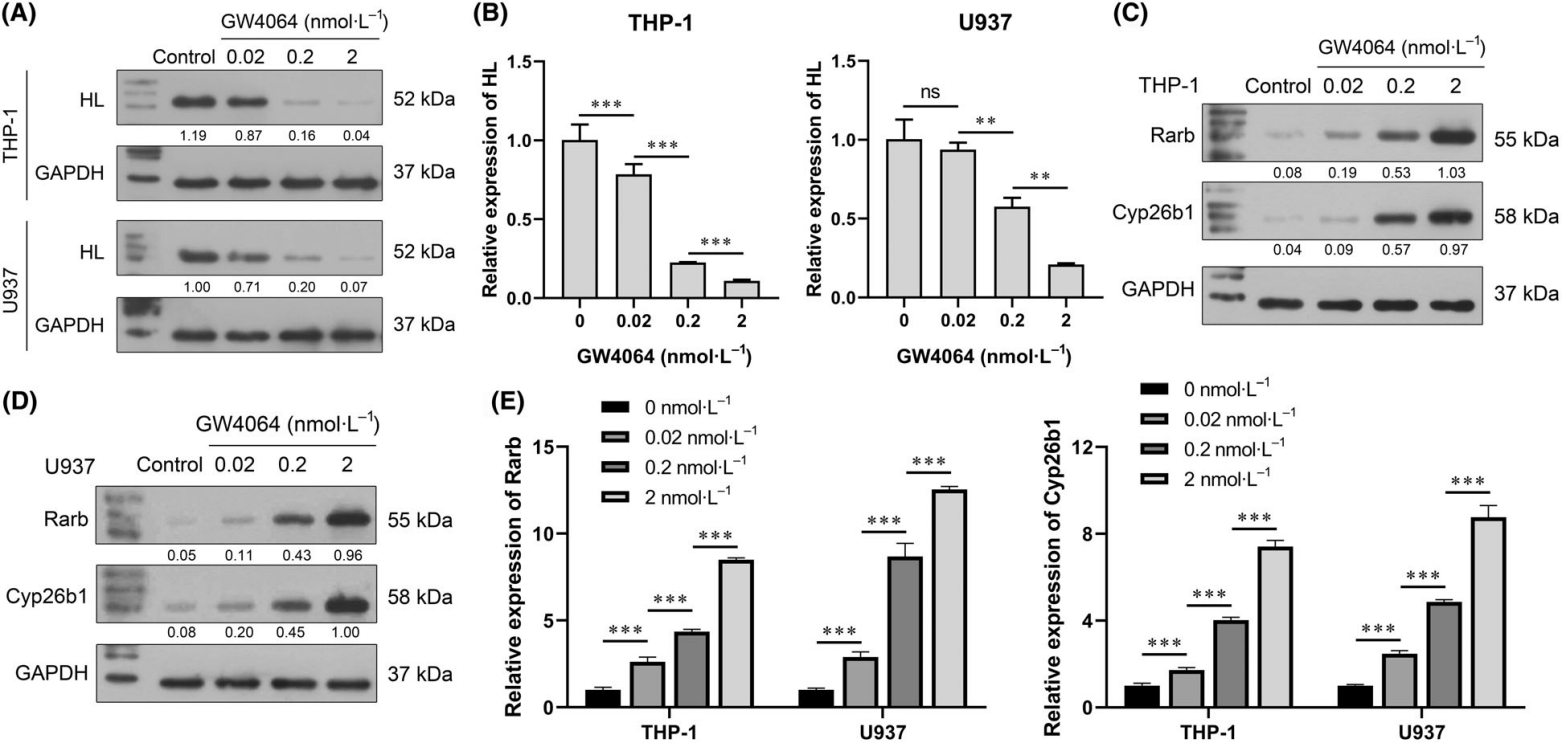
Effect of an FXR agonist on *FXR* transcription activity and HL expression. (A, B) HL protein and mRNA expression in GW4064‐treated macrophage‐derived foam cells were quantified by western blotting and qPCR (one‐way analysis of variance). (C–E) Rarb and Cyp26b1 protein and mRNA expression in GW4064‐treated macrophage‐derived foam cells were quantified by western blotting and qPCR (one‐way analysis of variance). ***P < 0.001; **0.001 < *P* < 0.01; value ± SD, one‐way analysis of variance; *n* = 3.

In this study, THP‐1 and U937 cells, which are well‐characterized human promonocytic cell lines, were used as model systems in which to study the mechanisms of macrophage‐derived foam cell formation induced by ox‐LDL in atherosclerosis. As shown in Fig. 2A, PMA successfully induced THP‐1 and U937 cells to differentiate into macrophages. Foam cell formation induced by ox‐LDL was confirmed by Oil Red O staining, which showed higher levels of lipid accumulation in the PMA + ox‐LDL group than in the PMA group (Fig. 2B). Next, the effect of FXR on macrophage polarization was detected by flow cytometry after treatment with an FXR agonist (GW4046, 2 nmol·L^−1^). Polarization to M2 macrophages was confirmed by a decrease in the M1 biomarker CD86 and an increase in the M2 biomarker CD206 as detected by flow cytometry (Fig. 2C). Decreased levels of iNOS and IL‐6 expression, increased levels of Arg1 and IL‐10 expression also proved the transition of M1 to M2 macrophages (Fig. 2D,E). Next, the effects of GW4046 on foam cell formation and lipid accumulation were determined by Oil Red O staining. Our results showed that GW4064 inhibited lipid accumulation in a dose‐dependent manner (Fig. 2F–H). Furthermore, qPCR and western blot studies confirmed that GW4064 inhibited HL expression at both the RNA and protein levels (Fig. 3A,B). We also detected the downstream genes (*Rarb* and *Cyp26b1*) of FXR, and the results showed that they were overexpressed after treatment with GW4064, which also confirmed that FXR was activated by GW4064 (Fig. 3C–E).

### FXR activation decreased serum cholesterol and triglyceride levels in ApoE^−/−^ mice

Next, effect of FXR was explored *in vivo*. WT C57 BL/6 mice and ApoE^−/−^ mice were fed either a NFD or HFD and then treated with GW4064. The levels of serum TC, TGs, and LDL‐C were measured and are shown in Table 1. After 8 weeks of consuming a HFD, the ApoE^−/−^ mice showed dyslipidemia, as indicated by marked increases in plasma TC, TG, and LDL‐C levels, as compared to C57 BL/6 mice fed a NFD. Moreover, this dyslipidemia was significantly alleviated by consumption of a GW4064‐supplemented diet by gavage. More specifically, at the end of week 8, both the HFD + 10 mg·kg^−1^ GW4064 group and HFD + 20 mg·kg^−1^ GW4064 group had decreased levels of plasma TC, TGs, and LDL‐C, when compared with mice in the HFD + 0 mg·kg^−1^ GW4064 group.

**Table 1:** TC, TG, and LDL‐C levels in mouse serum of all groups. Value ± SD, Student's *t*‐test.

Group	*n*	TC (mmol·L^−1^)	TG (mmol·L^−1^)	LDL‐C (mmol·L^−1^)
NFD + C57 BL/6 mice ApoE^−/−^ mice	10	5.56 ± 0.64	3.78 ± 2.52	6.54 ± 3.12
HFD + 0 mg·kg^−1^ GW4064	10	64.32 ± 2.07^a^	25.89 ± 3.05^a^	16.12 ± 2.16^a^
HFD + 10 mg·kg^−1^ GW4064 group	10	45.83 ± 5.67^b^	14.04 ± 3.23^b^	6.02 ± 5.81^b^
HFD + 20 mg·kg^−1^ GW4064 group	10	36.80 ± 7.04^b^, ^c^	5.48 ± 3.55^b^, ^c^	8.67 ± 2.78^b^

^a^ *P* < 0.01, compared with the NFD + C57 BL/6 mouse group

^b^ *P* < 0.01, compared with the HFD + 0 mg·kg^−1^ GW4064 group

^c^ *P* < 0.01, compared with the HFD + 10 mg·kg^−1^ GW4064 group.

### FXR activation regulated HL expression in macrophages to hinder atherosclerosis development in ApoE^−/−^ mice

**Fig. 4:**
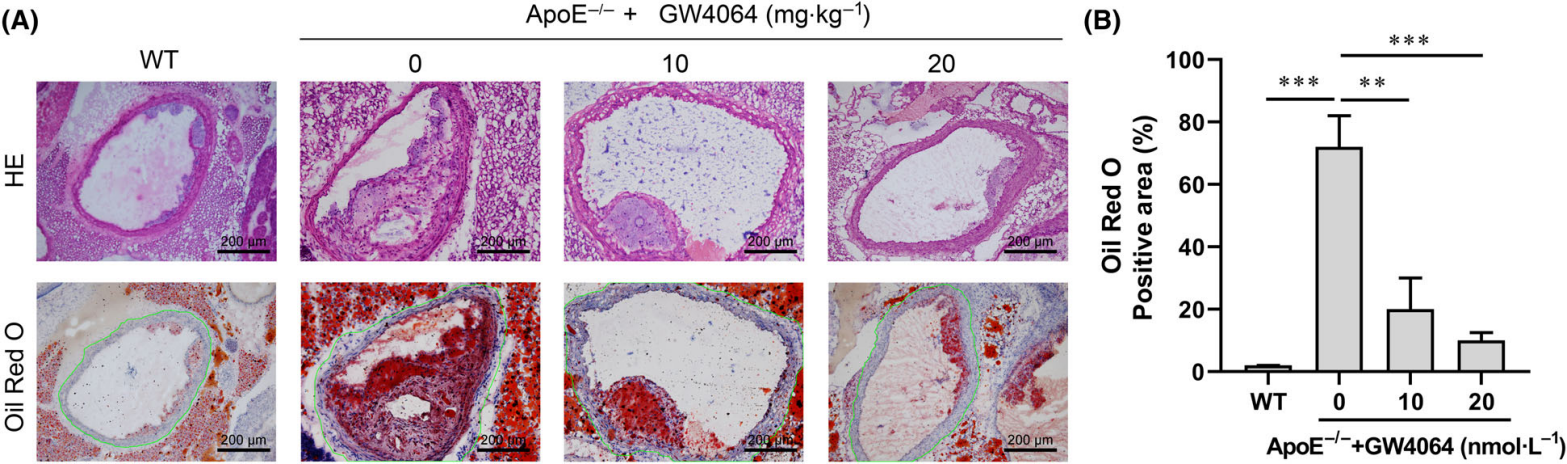
GW4064 alleviated atherosclerotic plaque formation in ApoE^−/−^ mice. (A) Representative images of H&E staining and Oil red O staining (magnification, ×200) of aortic cross sections from normal chow‐fed C57/BL6 mice (WT) and HFD‐fed ApoE^−/−^ mice treated with different doses of GW4046. (B) The positive area of oil‐red staining in ROI (green drawing area) was quantified (one‐way analysis of variance). ***P < 0.001; **0.001 < *P* < 0.01; value ± SD, one‐way analysis of variance; *n* = 10.

**Fig. 5:**
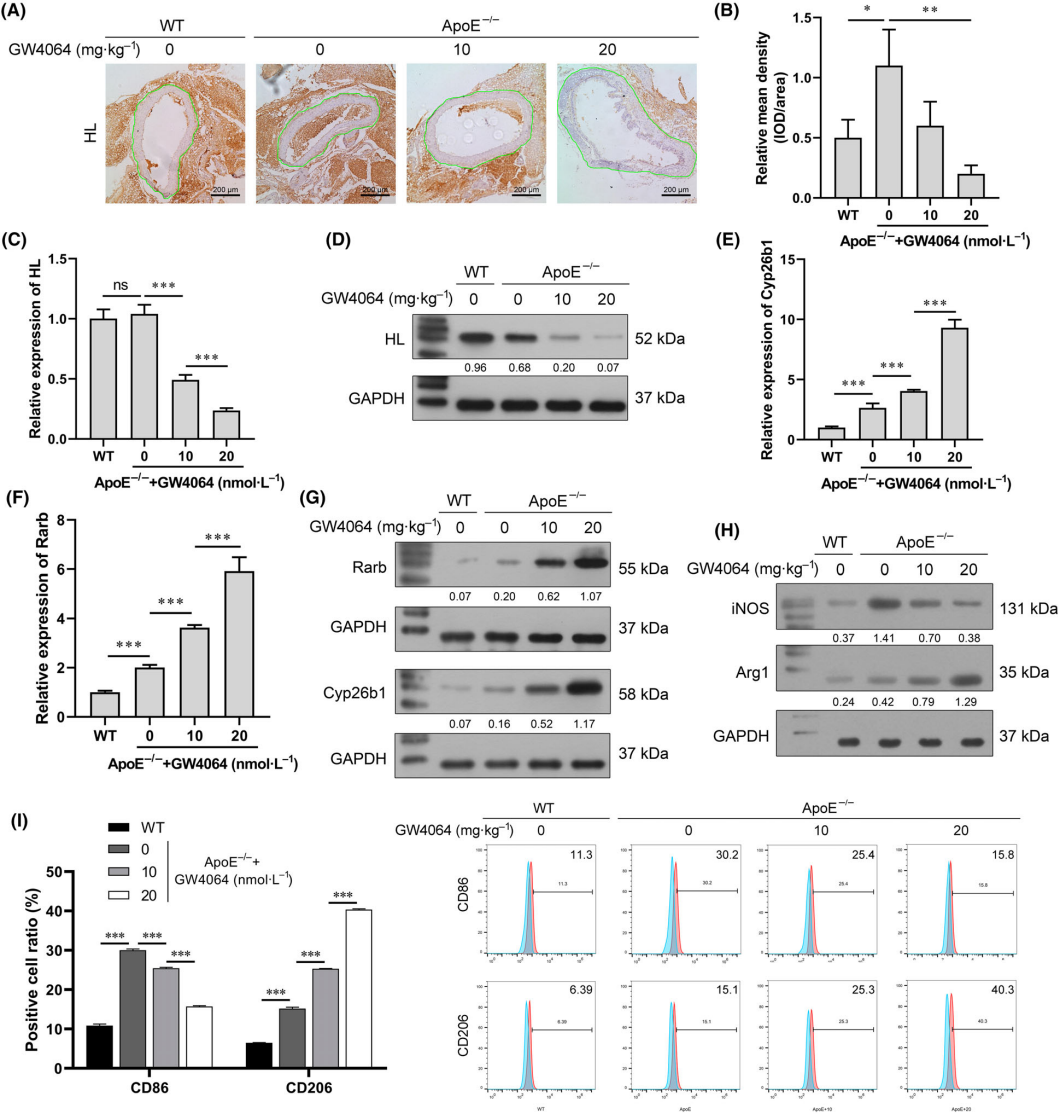
GW4064 downregulated HL expression. (A) Immunohistochemical staining was employed to detect the expression of HL in ApoE^−/−^ mice treated with different doses of GW4046 (magnification ×200). (B) The quantitative analysis of HL IHC staining in ROI (green drawing area) (one‐way analysis of variance). (C, D) Expression of HL in macrophages was detected by qPCR and western blotting (E–G) Expression of Rarb and Cyp26b1 in macrophage was detected by qPCR and western blot assay (one‐way analysis of variance). (H) The M1 macrophage marker iNOS and M2 macrophage marker Arg1 were detected by western blotting (I) The effect of GW4064 on macrophage polarization was detected by flow cytometry (one‐way analysis of variance). ns, no significant difference; *** *P* < 0.001; **0.001 < *P* < 0.01; *P < 0.05; value ± SD, one‐way analysis of variance; *n* = 10.

Oil Red O and H&E staining showed that GW4064 could inhibit the formation of AS plaque (Fig. 4A,B). At the same time, HL protein expression in the aorta wall was obviously decreased after GW4064 intervention (Fig. 5A,B). An analysis of plaque in the aortic arches showed that GW4064, as a potent agonist for the nuclear receptor FXR, most likely exerted its anti‐atherosclerotic effects and alleviated atherosclerotic plaque formation in the ApoE^−/−^ model mice by regulating HL expression. In primary peritoneal macrophages isolated from the ApoE^−/−^ mice, our data showed that HL expression was decreased by GW4064 (Fig. 5C,D), while Rarb and Cyp26b1 expression were increased by GW4064 (Fig. 5E–G). A decrease in iNOS expression and increase in Arg1 expression demonstrated that GW4064 promoted the transition of M1 to M2 macrophages (Fig. 5H). Moreover, flow cytometry assays confirmed the M2 polarization of macrophages by GW4064 (Fig. 5I). Taken together, these data showed that the synthetic FXR agonist GW4064 exerted anti‐atherosclerotic effects, possibly by downregulating HL expression in macrophages.

## Discussion

FXR, as a member of the nuclear receptor superfamily, has attracted widespread attention in the biomedical field in recent years. This receptor plays a central role in regulating bile acid, cholesterol, and lipid metabolism, and its dysfunction is closely related to a variety of disease states, such as fatty liver [21], cholesterol calculus [22], and atherosclerosis [23]. As research on the mechanism of FXR has increased, its potential for use in drug development has gradually emerged [24]. Our previous study identified *HL* as novel FXR‐regulated genes [12]. The present study was a follow‐up to our previous study about the mechanism that treatment of human hepatic cells with an FXR agonist downregulated the HL transcription levels, by which bile acids modulate triglyceride, fatty acid, and HDL metabolism and further support the central role of FXR in lipid homeostasis. The present study showed that GW4064‐activated FXR mediated a dose‐dependent downregulation of *HL* transcription in THP‐1 and U937 cells and peritoneal macrophages in ApoE^−/−^ mice. Moreover, our data from transient transfection experiments revealed a FXR‐binding site in human U937 monocytic cells located in the −217 to −196 bp fragment of the human *LIPC* gene promoter. Thus, FXR might directly regulate *LIPC* gene transcription. The downregulation of HL by an FXR agonist suggests a novel mechanism that may contribute to the modulation of HDL and triglyceride metabolism by FXR.

Hepatic lipase is crucial for reversing cholesterol transport and plays important roles in modulating the metabolism and serum levels of several lipoproteins [25, 26]. HL is mainly synthesized and secreted by the liver [27]. Our results showed that downregulation of HL by GW4064 was related to decreased foam cell formation *in vitro*. We also found altered macrophage polarization in ApoE^−/−^ mice treated with GW4064, accompanied by decreased atherosclerotic plaque formation, which also suggests the potential role of HL. Interestingly, we found that the *HL* gene was also down‐expressed in macrophages derived from THP‐1 and U937 cells after GW4064 stimulation. It can be speculated that a regulatory relationship between FXR and HL exists in foamy macrophages.

Macrophages play a pivotal role in the initiation, progression, and complications of atherosclerosis. These immune cells infiltrate the arterial wall, transform into foam cells, and secrete inflammatory mediators that promote plaque growth and instability [28]. Ox‐LDL‐mediated foam cell formation may be related to lipid rafts and oxidation processes [29]. In this study, inhibition of macrophage foam cell formation induced from THP‐1 and U937 cells also showed decrease lipid accumulation. When considering the role of FXR in inhibiting lipid peroxidation [9], it is easy to understand why activation of FXR can inhibit foam cell formation. Another important finding was that activation of FXR affected macrophage polarization. In the study, we found that activation of FXR decreased the numbers of CD86^+^ cells and increased the numbers of CD206^+^ cells. A previous study found that ox‐LDL promotes M1 macrophage polarization [30]. Similar to our results, Thiranut Jaroonwitchawan *et al*. [31] reported that an FXR agonist activated polarization toward M2 but not M1 macrophages. In addition, hepatic lipase‐deficiency in mice was found to promote macrophage‐to‐feces HDL antioxidant properties [32]. Besides M1 and M2, other macrophage polarization phenotypes also participate in atherosclerosis. One of those phenotypes, designated as the M4 macrophage, can be induced by CXCL4 [33] and inhibit expression of the anti‐atherosclerosis enzyme HO‐1 [34]. Another phenotype M(Hb) is induced by hemoglobin and prevents foam cell formation from macrophages [35]. Moreover, Mox, which is induced by atherogenic phospholipids, shows a characteristically strong antioxidant capability [36]. These phenotypes may also play an important role in atherosclerosis and should be further investigated.

Our IHC results (Fig. 5A) showed that the staining density of HL was reduced by GW4064, both in blood vessels and surrounding tissues, indicating that GW4064 could directly affect blood vessel cells. Therefore, regulation of macrophage foam cell formation may not be the only way that GW4064 and FXR affect atherosclerotic plaque. In fact, FXR and GW4064 have been reported to function in vascular reactivity by regulating the calcium homeostasis of aortic vascular smooth muscle cells (VSMCs) [37, 38]. Other studies have also demonstrated that FXR and GW4064 regulate the proliferation, migration, and inflammation of VSMCs [39, 40]. Collectively, GW4064 may reduce atherosclerotic plaque formation by directly affecting blood vessels, and this hypothesis deserves to be studied.

**Fig. 6:**
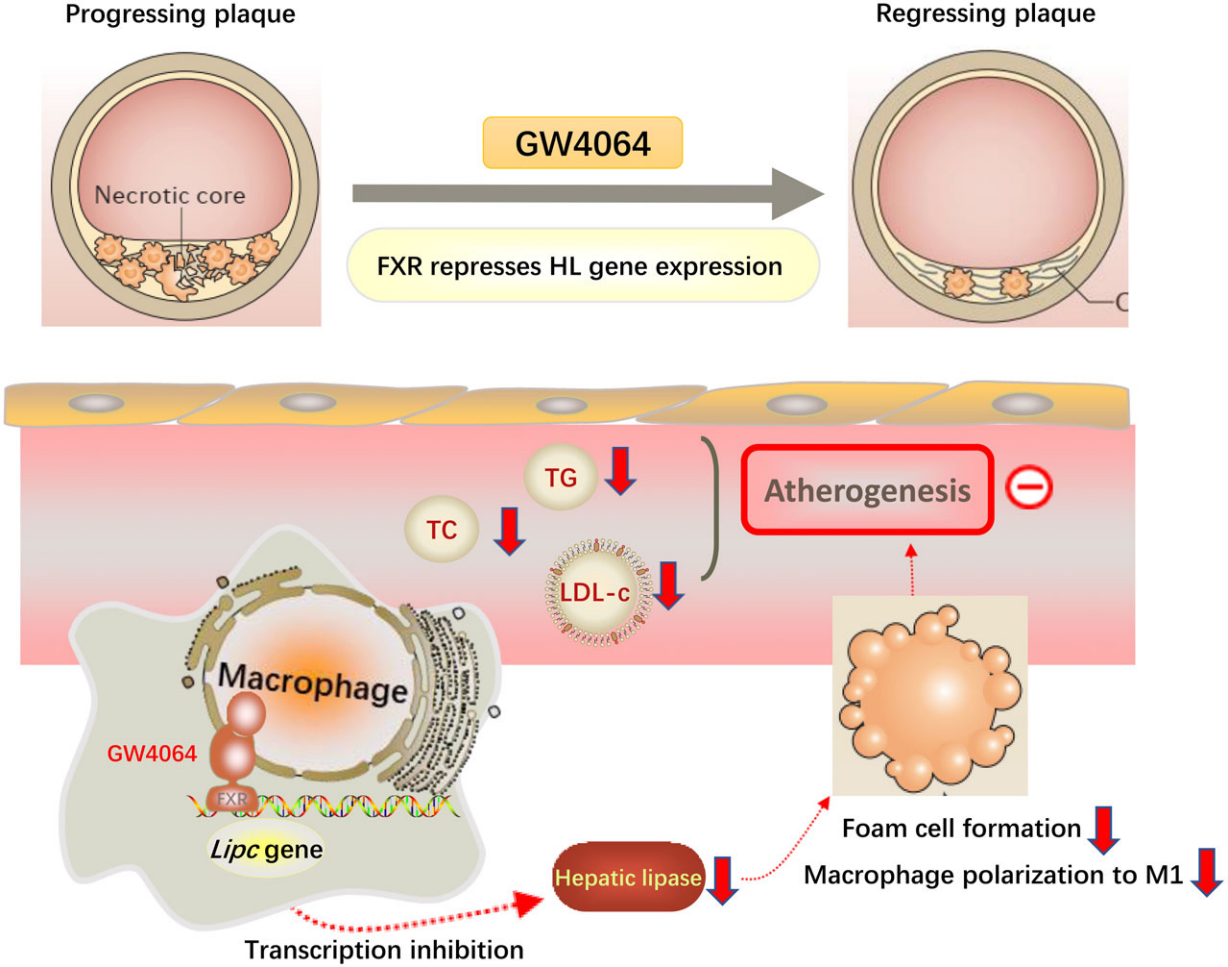
The FXR agonist GW4064 exerted anti‐atherosclerotic effects by regulating *LIPC* transcription. GW4064 activated FXR to inhibit *LIPC* transcription, then decreased HL inhibits foam cell formation and polarization to M1 macrophages, thereby reducing the production of atherosclerotic plaque and suppressing atherogenesis.

In summary, our results suggest that activation of FXR by GW4064 produces anti‐atherosclerotic effects that result from a combined modulation of lipid metabolism and macrophage foam cell formation (Fig. 6), which may exert their function by regulating *LIPC* transcription. Our data suggest that FXR ligands and HL may have utility in the treatment of atherosclerotic disorders.

## Conflict of interest

The authors declare no conflict of interest.

### Peer review

The peer review history for this article is available at https://www.webofscience.com/api/gateway/wos/peer‐review/10.1002/2211‐5463.13925.

## Author contributions

QG performed most of the molecular biology and genomic experiments, analyzed the data, and drafted the manuscript. JL performed some of the animal experiments and analyzed the data. LLS performed some of the immunofluorescence experiments. QG and LLS developed the concept, designed the study, and wrote the manuscript.

## Supporting information


**Table S1.** Collection of FXR binding sites on the human HL promoter.
**Table S2.** Primers used for EMSA.
**Table S3.** PCR Primers used for THP‐1 cells and ApoE^−/−^ & C57BL/6 mice.
**Table S4.** TC, TG and LDL‐C levels in mouse serum of all groups.

## Data Availability

All data are available from the published article.
