# Supplementary material for: FXR activation reduces the formation of macrophage foam cells and atherosclerotic plaque, possibly by down regulating hepatic lipase in macrophages
Source: FEBS Open Bio. 2024 Nov 27;15(2):311–23. doi: 10.1002/2211-5463.13925 (PMC11788749; doi:10.1002/2211-5463.13925)
Supplement: Supplementary file 1 — Table S1. Collection of FXR binding sites on the human HL promoter. Table S2. Primers used for EMSA. Table S3. PCR Primers used for THP‐1 cells and ApoE−/− & C57BL/6 mice. Table S4. TC, TG and LDL‐C levels in mouse serum of all groups. [file FEB4-15-311-s001.docx]

**Table S1. Collection of FXR binding sites on the human HL promoter**

| Repeat | Position(strand) | Score | Z-score | P value | E value | Half-site E value | Site sequence |
| --- | --- | --- | --- | --- | --- | --- | --- |
| ER10 | 1684(+) | 0.861562 | n/a | 0.00193297 | n/a | n/a | TGACCTtggccccaaaAGGTAA |
| ER6 | 1340(+) | 0.84532 | n/a | 0.00228161 | n/a | n/a | TCACCTctcaatGGGTCA |
| DR2 | 971(-) | 0.824653 | n/a | 0.0073496 | n/a | n/a | GGATCAcgAGGTCA |
| DR0 | 1695(-) | 0.824653 | n/a | 0.0073496 | n/a | n/a | GGGCCAAGGTCA |
| DR7 | 1352(+) | 0.821024 | n/a | 0.00296734 | n/a | n/a | GGGTCActtggcaAGGGCA |
| DR9 | 1337(+) | 0.802283 | n/a | 0.00393573 | n/a | n/a | GGATCAcctctcaatGGGTCA |

**Table S2. Primers used for EMSA**

| Targets | Primers |
| --- | --- |
| U937 cells  labeled probe |  |
| WT-ER10-F:  WT-ER10-R: | 5’-AACTC**TGACCTTGGCCCCAAAAGGTAA**GAATA-3’  5’- TATTCTTACCTTTTGGGGCCAAGGTCAGAGTT-3’ |
| Mut- ER10-F:  Mut- ER10-R: | 5’-AACTC**TGgtCTTGGCCCCAAAAGacAA**GAATA-3’  5’- TATTCTTGTCTTTTGGGGCCAAGACCAGAGTT -3’ |
| Unlabeled cold probe  WT-ER10-F:  WT-ER10-R: | 5’- AACTC**TGACCTTGGCCCCAAAAGGTAA**GAATA-3’  5’- TATTCTTACCTTTTGGGGCCAAGGTCAGAGTT-3’ |
| Mut- ER10-F:  Mut- ER10-R: | 5’- AACTC**TGgtCTTGGCCCCAAAAGacAA**GAATA-3’  5’- TATTCTTGTCTTTTGGGGCCAAGACCAGAGTT -3’ |

Boldface letters indicate transcription factor binding regions, and lowercase letters show mutated bases

**Table S3. PCR Primers used for THP-1 cells and ApoE ^-/-^& C57BL/6 mice.**

| Targets | Primers |
| --- | --- |
| THP-1 and U937 cells |  |
| human HL | F-5′- ATCAAGTGCCCTTGGACAAAG -3′  R-5′- TGACAGCCCTGATTGGTTTCT - 3′ |
| human Rarb | F-5′- CCAGCAGTGAACGGACACAT -3′  F-5′- TCCTCCGATGACAGGTGGAA -3′ |
| human Cyp26b1 | F-5′- CGCCACTCGCGACAAG -3′  F-5′- AATGCGTCTTGAACACGTTG -3′ |
| human GAPDH | F-5′- GACATCAAGAAGGTGGTGAA -3′  R-5′- CCACATACCAGGAAATGAGC -3′ |
|  |  |
| ApoE -/-& C57BL/6 mice |  |
| murine HL | F-5′- TGGATCTGGAAGATAGTGAGTGCGCTGAA -3′  R-5′- CTGTGATTCTTC-CAATCTTGTTCTTCCCG - 3’ |
| murine Rarb | F-5′-AAGCTTGAATGCAGCCATCG -3′  F-5′-CTTGTCCTGGCAAACGAAGC -3′ |
| murine Cyp26b1 | F-5′-AAGACACACTTACTGGGGCG -3′  F-5′-CTCAAGTGCCTCATGGCTGA -3′ |
| murine GAPDH | F-5′- CTGAGTATGTCGTGGAGTCTAC -3′  R-5′- GTTGGTGGTGCAGGATGCATTG -3′ |

**Table S4. TC, TG and LDL-C levels in mouse serum of all groups**

| Group | n | TC（mmol/L） | TG（mmol/L） | LDL-C（mmol/L） |
| --- | --- | --- | --- | --- |
| NFD+C57 BL/ 6 mice  ApoE-/- mice | 10 | 5.56 ±0.64 | 3.78 ±2.52 | 6.54±3.12 |
| HFD+0 mg/kg GW4064 | 10 | 64.32 ±2.07^a^ | 25.89 ±3.05^a^ | 16.12±2.16^a^ |
| HFD+10 mg/kg GW4064 group | 10 | 45.83±5.67 | 14.04 ±3.23 | 6.02 ±5.81 |
| HFD+20 mg/kg GW4064 group | 10 | 36.80±7.04^b^ | 5.48±3.55^b^ | 8.67±2.78^b^ |

a.P<0.01, compared with the NFD+C57 BL/ 6 mice group; b. P<0.01, compared with the HFD+0 mg/kg GW4064 group.
